# Coblation debulking of a paediatric laryngeal plexiform neurofibroma: a pragmatic response to a rare tumour

**DOI:** 10.1093/jscr/rjab646

**Published:** 2022-01-26

**Authors:** James Schuster-Bruce, Mairead Kelly, Ana Bernic, Sabrina Brar, Joy Barber, Prince Modayil

## Abstract

Laryngeal neurofibroma is a rare but important differential diagnosis in a patient presenting with stridor. In paediatric patients, these lesions present a management conundrum: complete surgical resection is the established treatment of choice, but an aggressive approach can be detrimental to developing anatomy. We report the case of a plexiform neurofibroma affecting the right hemilarynx of a 3-year-old boy. Endoscopy revealed a large tumour, involving the right aryepiglottic fold and extending into the piriform sinus, ventricle and the false cord. Given the patient’s young age and the challenging tumour location, the lesion was debulked, rather than resected, using coblation (low-temperature plasma radiofrequency ablation). At 30 months follow-up, the neurofibroma has mildly increased in size—in line with expectations that these lesions exhibit slow growth throughout childhood—but there are no significant respiratory symptoms and there is no functional impairment.

## INTRODUCTION

Neurofibromatosis type 1 (NF1) is an autosomal dominant condition affecting 1 in 3000 births [1]. Neurofibromatosis of the larynx is rare, accounting for 0.03–0.1% of benign laryngeal tumours [2, 3]. Neurofibromas are classified as plexiform, diffuse or both, depending on growth pattern [4]. Although there are over 60 case reports of paediatric laryngeal neurofibromas [5, 6], the majority describes complete surgical resection [7–10]. Fewer describe less invasive measures—and only two used coblation [5, 11]; however, both of these describe its use for complete resection, rather than for debulking: thus we believe this report to be the first of its kind.

## CASE REPORT

A 3-year-old boy presented to a district general hospital with acute airway obstruction. His medical history included NF1, low grade cervico-medullary astrocytoma and epilepsy with absence seizures.

**Figure 1:**
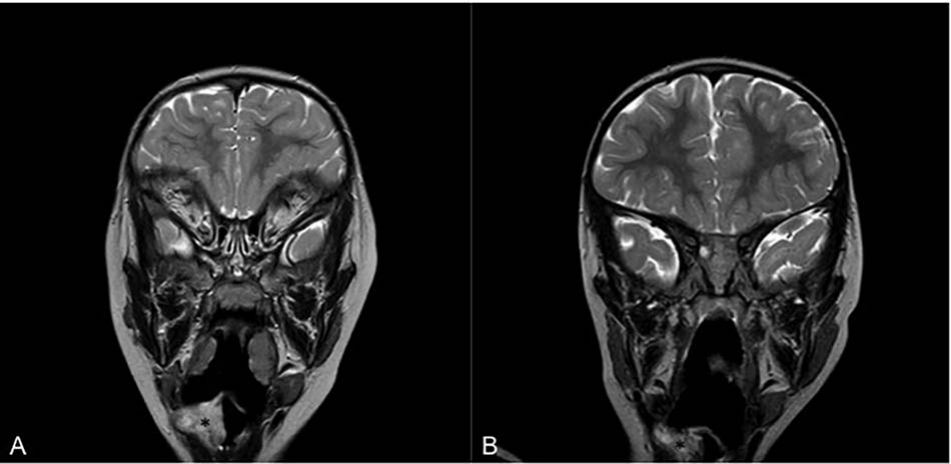
Magnetic resonance imaging (MRI) of head and neck with contrast in sagittal view on admission (**A**) and at 16 months follow-up (**B**). Asterisk denotes area of interest.

Flexible nasoendoscopy found a 1 × 1.5 cm sessile mass adjacent to the epiglottis, also seen on magnetic resonance imaging (MRI) (Fig. 1A). The patient was referred to a tertiary Ear, Nose and Throat (ENT) centre and admitted to the paediatric intensive care unit.

**Figure 2:**
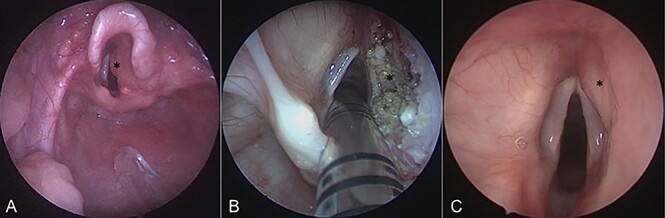
Intraoperative endoscopic images of the supraglottis: (**A**) before intervention; (**B**) after debridement; and (**C**) at 16 months after surgical intervention. Asterisk denotes area of interest.

On admission, his oxygen saturations, serum haematology and biochemistry were normal. There was no daytime respiratory distress. At night, he had audible inspiratory stridor, a raised respiratory rate, tracheal tug and apnoeas. He underwent a diagnostic microlaryngoscopy, which showed a large, submucosal, nodular tumour involving the right aryepiglottic fold, false cord, ventricle and the medial wall of the piriform fossa (Fig. 2A). Histological analysis of biopsies described multiple tortuous nerve fascicles; the clinical suspicion of a plexiform neurofibroma was confirmed.

The case was discussed in the Head and Neck multi-disciplinary team (MDT) meeting. Due to the patient’s age, co-morbidities and the site of the lesion, the MDT felt that complete surgical excision—requiring resection of the right aryepiglottic fold, false cord and ventricle—would result in significant morbidity. The child would be at risk of aspiration, dysphagia, dysphonia and laryngeal scarring. Therefore, we opted to use a coblation technique to debulk the neurofibroma (Fig. 2B).

Post-operatively, the number of observed apnoeic episodes improved significantly. However, intermittent noisy breathing at night was ongoing, so, 3 months later, the patient underwent a further debridement, as well as coblation intracapsular tonsillotomy and adenoidectomy. Following this, his symptoms improved considerably, with noisy night-time breathing resolving, and serial sleep studies showing significant improvement. He did not experience any dysphagia or aspirations, confirmed by a review from a speech and language therapist.

Sixteen months after the second debridement, the patient had a largely asymptomatic airway, stable disease on MRI scans (Fig. 1B) and no signs of significant lesional growth or airway obstruction on repeat microlaryngoscopy (Fig. 2C). At 30 months follow-up, imaging shows the neurofibroma to have mildly increased in size but there are no significant respiratory symptoms and no functional impairment.

## DISCUSSION

Although there are more than 60 case reports of paediatric laryngeal neurofibroma [5, 6], the majority describe complete surgical resection as the gold standard treatment [7–10]. Both an open, cold steel surgical approach and, more recently, endoscopic C02-laser have been reported in the literature [3, 12–16]. An extreme case of a total laryngectomy in a 5-year-old female is also reported [17]. Our submission is that there are pragmatic, safe alternatives—such as coblation—which allow debulking of a tumour and provide long-term symptomatic relief, while preserving the developing laryngeal structures.

Although complete resection is desirable, it has attendant risks when operating on neurofibromas of the larynx. The larynx is functionally varied, and therefore excision of a laryngeal lesion can carry significant morbidity including dysphonia, aspiration and scarring. A further concern is the increased need for tracheostomy [6]. These are particularly pertinent considerations in the paediatric population, where the operation has greater technical difficulty, the anatomical structures are still developing and there is an increased risk of psychological trauma. The plexiform subtype is also more challenging to excise fully [18].

There is much sparser literature on the use of less aggressive techniques—indeed there are only two other case reports which describe coblaton for this indication [5, 11]. In both of these cases, the authors describe ‘complete removal’ of tumours. Wang *et al*.’s description has more in common with a debulking technique (preceded by a tissue biopsy taken with forceps) [5], whereas Pradhan *et al.* excised the mass en bloc ‘with the help of endoscopic coblation’ [11].

Other methods that could be considered for debulking a tumour like this are C02-laser, a micro-debrider and cryotherapy [6, 19, 20]. In this case, micro-debrider surgery and cryotherapy were dismissed due an increased risk of trauma to surrounding tissues and a lack of evidence, respectively [20]. We chose coblation because it has a long track record as an operative modality, is widely available, and allows a bloodless field [21, 22]. It is also used to treat neurofibromas in other anatomical sites [23]. It creates high-energy flux to ablate tissue, while maintaining a low thermal energy of 40–60°C [21]. This reduces thermal damage to surrounding tissues, the risk of subsequent scarring and the risk of airway fire—all recognized complications of trans-oral C02 laser, which operates at higher temperatures.

A disadvantage of using this technique for debulking, rather than excision, is that complete lesional resection cannot be achieved. Without this, it is possible for the neurofibroma to re-grow and to undergo malignant transformation. Reassuringly, the plexiform subtype is rarely malignantly invasive and is typically slow-growing [18, 24]. The growth rate is fastest in childhood, so children may require further biopsies and debulking procedures to differentiate fast benign growth from malignant transformation, particularly in the context of growth ‘spurts’. For this reason, regular follow-up of these patients is required. In the present case, there was no evidence of disease progression at 16 months, and in the only comparable case report, by Wang *et al*., the same was true at 2 years [5]. In the current case, at 30 months’ follow-up, the neurofibroma has mildly increased in size—in line with expectations that these lesions exhibit slow growth throughout childhood—but there are no significant respiratory symptoms and no functional impairment. We submit, therefore, that despite the limitations, given the technique’s advantages, coblation debulking provides a pragmatic approach to the management of paediatric laryngeal plexiform neurofibromas.
